# Microwave Thermal Ablation for Breast Cancer in Africa: A Pioneering Case Report Utilizing TATOpro

**DOI:** 10.7759/cureus.64029

**Published:** 2024-07-07

**Authors:** George Asafu Adjaye Frimpong, Emmanuel Asante, Fairuuj Mahama, Evans Aboagye, Adwoa Asare

**Affiliations:** 1 Radiology, Kwame Nkrumah University of Science and Technology, Kumasi, GHA; 2 Radiology, Spectra Health Imaging and Interventional Radiology, Kumasi, GHA; 3 Research and Development, Spectra Health Imaging and Interventional Radiology, Kumasi, GHA; 4 Oncology, Komfo Anokye Teaching Hospital, Kumasi, GHA

**Keywords:** africa, minimally invasive, coagulation, breast cancer, microwave ablation

## Abstract

The adoption of minimally invasive treatments for early-stage breast cancer is increasing. Microwave thermal ablation (MWA), a minimally invasive technique, has been studied for treating small breast cancer lesions. However, long-term evidence on its efficacy as a sole treatment is limited, as most studies combine MWA with other therapies and post-treatment surgical excision. This report details the case of an 83-year-old African patient who declined surgery and systemic therapies, opting for MWA using the TATOpro system as the sole treatment for contralateral breast cancer with axillary lymph node metastasis. The report includes a one-year follow-up, assessing disease recurrence with MRI and ultrasound. The findings highlight MWA's potential as an innovative and efficacious breast cancer treatment, emphasizing the need for adaptable strategies in oncology.

## Introduction

Over the years, surgical treatment options for breast cancer have evolved from mastectomy to lumpectomy to enhance post-surgical aesthetics, reduce morbidity, and enhance the quality of life without compromising treatment effectiveness [1,2]. Interestingly, inoperable cancers, due to the age of patients, previous history of surgery, and patient preferences, have paved the way for more impactful studies on minimally invasive treatments [3].

Percutaneous microwave thermal ablation (MWA) is a relatively new thermal technology and a minimally invasive cancer treatment that applies microwave energy to heat targeted cancer tissues to threshold temperatures that cause immediate coagulative necrosis without destroying normal tissue [4]. During MWA, continuous monitoring is conducted using ultrasound. Additionally, recommended follow-up imaging for cancer treatment post-MWA includes positron emission tomography-computed tomography (PET-CT) and MRI scans. While follow-ups at one-month and three-month intervals after MWA are suggested for lung, renal, and liver cancer, there are no consensus guidelines on follow-up procedures for breast cancer treatment with MWA [5,6].

Residual tumors and local recurrence after thermal ablation are a great challenge for these minimally invasive treatments, and many studies have added them to chemotherapy and surgery to achieve better outcomes. Alone, MWA offers several advantages over other thermal techniques: it achieves higher intratumoral temperatures, creates larger ablation volumes of up to 66.5 cm^3^, and requires shorter ablation times [7]. Consequently, MWA has been effective in treating various malignancies, including liver, lung, kidney, and bone tumors [8,9]. However, the body of literature on percutaneous MWA in breast cancer treatment remains limited [10,11].

This report presents the first documented case of a sole MWA treatment of an 83-year-old woman with metachronous contralateral breast cancer in Africa, employing the TATOpro system and providing imaging insights into its preliminary clinical outcome.

## Case presentation

An 83-year-old woman with a positive family history of breast cancer presented with metachronous contralateral cancer of the left breast at Spectra Health Imaging and Interventional Radiology Center. Ten years prior, the patient presented with a lump in the right breast, for which a mammogram found a BI-RADS (Breast Imaging-Reporting and Data System) IV lesion. Biopsy and further immunohistochemistry confirmed a triple-negative invasive ductal carcinoma of no special type in the right breast with ipsilateral suspected lymph nodes and no metastatic disease (cT2N1M0). The patient was offered four cycles of Adriamycin at 60 mg/m^2 ^and cyclophosphamide at 600 mg/m^2^ as neoadjuvant chemotherapy with a good clinical response and underwent a right-modified radical mastectomy with level I and II axillary nodal clearance. She further received adjuvant radiotherapy and four cycles of paclitaxel as adjuvant chemotherapy. Subsequent follow-ups were uneventful until the patient noticed a new mass in the left breast and reported to our facility for a mammogram.

**Figure 1:**
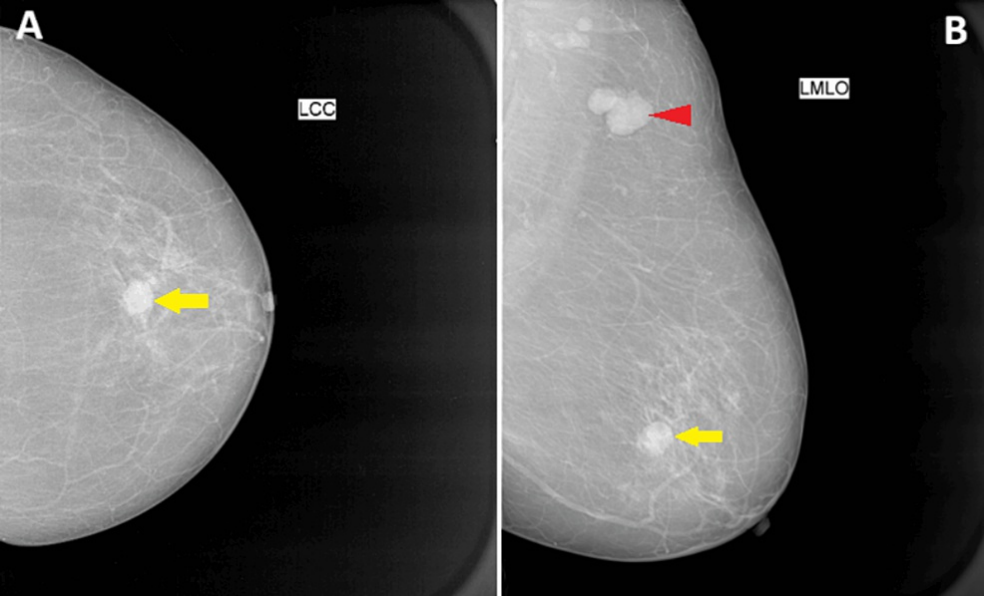
Mammogram (A) CC view showing relatively ill-defined opacity located at about 6 o'clock position of the left breast with subtle edge spiculation (arrow) and (B) MLO view showing a lobulated metastatic left axillary lymph node (arrowhead) CC: craniocaudal; MLO: mediolateral oblique

**Figure 2:**
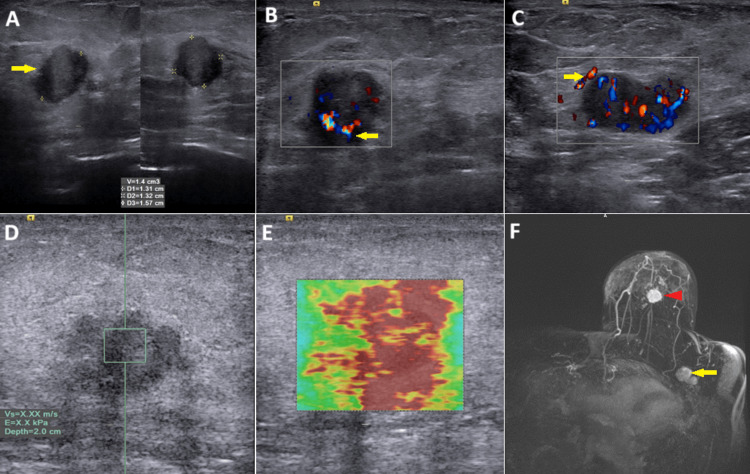
Pre-ablation images. (A) Greyscale ultrasound showing an irregularly edged hypoechoic lesion at about 6 o'clock position of the left breast (arrow). (B) Doppler ultrasound of the primary lesion showing moderate vascularity (arrow). (C) Greyscale ultrasound with color Doppler interrogation showing metastatic appearing left axillary lymph node with marked vascularity (arrow). (D) Quantitative ultrasound elastography showing very high stiffness above the default upper limit of SWE velocity (≥6.5 m/s) indicated as X.XX. (E) Qualitative ultrasound elastography showing very high stiffness evidenced by a predominately red color map. (F) Post-contrast dynamic MRI with subtraction MIP showing the primary lesion (arrowhead) and ipsilateral metastatic lobulated axillary lymph node (arrow) SWE: shear wave elastography; MIP: maximum intensity projection

Mammogram and ultrasound of the left breast showed a BI-RADS V lesion at about 6 o'clock position, measuring 1.5×1.5×1.5 cm, with a solitary lobulated ipsilateral axillary lymph measuring 2.0×1.0×1.5 cm (Figure 1 and Figure 2A). Moderate vascularity was observed for the primary lesion and axillary lymph nodes on color Doppler (Figure 2B, 2C). Ultrasound elastography of the primary lesion indicated malignant characteristics (Figure 2D, 2E). Post-contrast dynamic MRI with subtraction maximum intensity projection (MIP) showed a primary lesion and an ipsilateral metastatic lobulated axillary lymph node (Figure 2F). Biopsy confirmed imaging findings, and no other organ showed signs of metastatic disease on abdominal MRI or whole-body diffusion-weighted MRI.

MRI was performed with a 1.5 T system with 16 channels (MAGNETOM Essenza, Siemens, Germany) and dedicated breast coils. The patient refused radical surgical intervention or any systemic therapy and subsequently underwent an MWA treatment under ultrasound guidance. Under sterile conditions, a planning ultrasound was undertaken using the Siemens Acuson S3000 system and a 12 MHz high-frequency linear array transducer for lesion localization, needle trajectory, and procedure planning. The MWA procedure was performed in the interventional radiology room by two interventional radiologists with experience in over 1500 breast MRI interpretations. The patient received satisfactory local anesthesia with 5 mL of diluted lidocaine. An ultrasound-guided MWA was performed using a TATOpro system and an 18-gauge antenna (thinnest). A stab incision was made with surgical scalpel blade no. 11 at the same site where anesthesia was administered to the patient to facilitate the introduction of the MWA antenna needle. The power output was set at 15 W, and the procedure lasted 10 minutes. Repositioning the needle (18G) at intervals ensured that the entire lesion and the immediate perilesional 1 cm radius received adequate treatment. Subsequently, a track ablation using 20 W was performed to prevent tumor metastatic seeding and to stop bleeding before needle retraction. The ablation of the metastatic left axillary lymph node was not completed because the patient chose to discontinue, citing significant discomfort and shoulder joint pain even after proposing sedation or analgesia.

**Figure 3:**
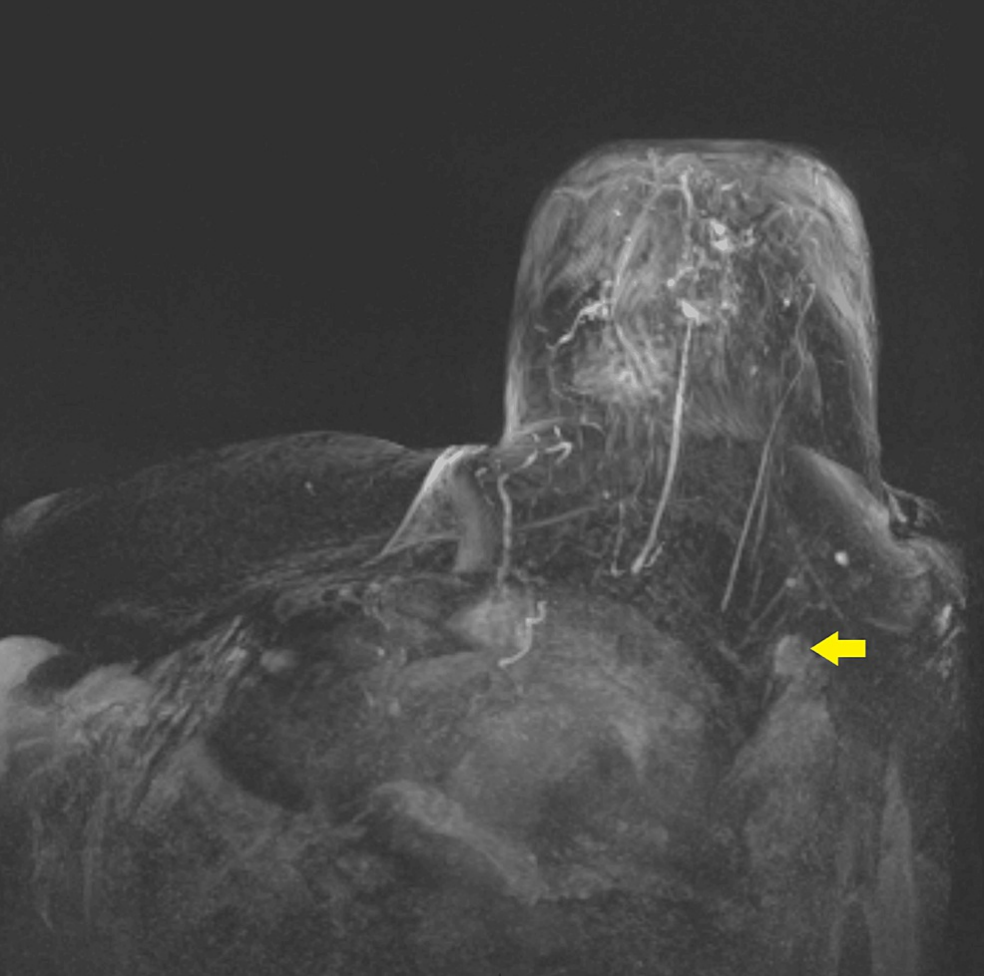
Two days post-ablation. Post-contrast dynamic MRI with subtraction MIP showing the absence of the primary lesion and a better outlined residual metastatic axillary lymph node (arrow) MIP: maximum intensity projection

**Figure 4:**
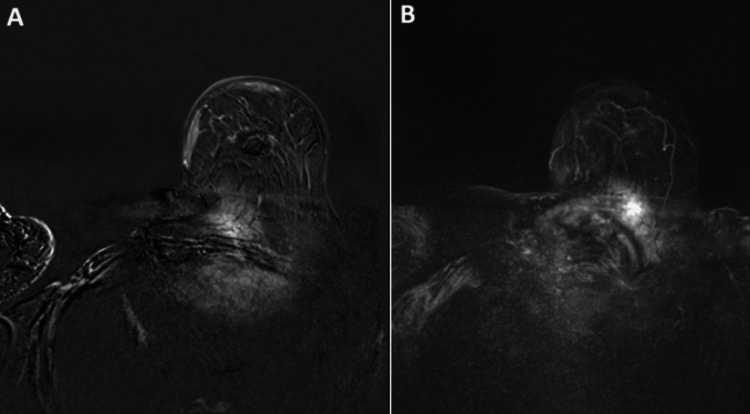
Seven months post-initial ablation. (A) T1 arterial phase showing ablated area with no suspicious abnormal enhancement. (B) Post-contrast dynamic MRI of the breast with MIP subtraction showing the continuous absence of the primary lesion and no residual ipsilateral axillary lymph node metastases after re-ablation MIP: maximum intensity projection

**Figure 5:**
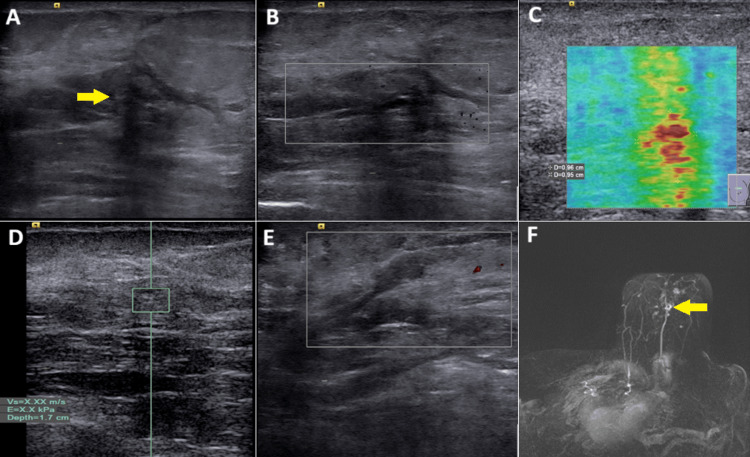
One year post-ablation. (A) Greyscale ultrasound showing an area of architectural distortion at the ablation site with no evidence of a tumor residue (arrow). (B) Color Doppler interrogation showing no vascularity. (C) Qualitative ultrasound elastography demonstrating an area of high stiffness (red) over the architectural distortion. (D) Quantitative ultrasound elastography showing very high stiffness above the upper limit (≥6.5 m/s), recorded as X.XX m/s. (E) Color Doppler interrogation showing no vascularity and architectural distortion for the metastatic appearing left axillary lymph node. (F) MRI showing focal fat necrosis in the region of the ablated region (arrow) and the absence of the left axillary lymph node metastasis

An MRI was performed two days post-ablation (Figure 3). A contrast agent bolus intravenous injection of 0.1 mmol of gadodiamide (OMNISCAN™) per kilogram of body weight was administered, followed by a 5 ml saline solution. After the contrast material was administered, the successfully ablated tumor was identified as a nonperfused volume. After two months, the patient agreed to a second MWA for the partially ablated metastatic ipsilateral axillary lymph node. Figure 4 shows images at seven months post-initial ablation of the primary and lymph node lesions. One year post-initial ablation, MRI, ultrasound, and elastography were performed to confirm the complete ablation of the primary tumor, ipsilateral axillary lymph node, and absence of recurrence of the disease (Figure 5).

## Discussion

Radiofrequency ablation, MWA, and cryoablation are promising thermal techniques currently being studied as alternatives to surgical resection of tumors with better cosmetic outcomes [6,12]. Especially, MWA has proven effective for treating benign breast lesions [13]. However, the current body of literature regarding the use of MWA for breast cancer treatment is limited, and there is no relevant data on its potential as a standalone breast cancer treatment option. We first report a successful experience of MWA in breast cancer in Africa utilizing the TATOpro system without tumor resection and adjuvant therapy. We also provide imaging insights for a one-year follow-up without disease recurrence.

In our case, complete clinical response, defined as the complete disappearance of all radiologically detectable malignant diseases, was achieved for both the primary lesion and the metastatic axillary lymph node. Studies by Zhou et al. and van de Voort et al. have reported rates of complete ablation of small breast cancers as high as 95% with few complications and less hospitability compared to surgery [6,14]. The complications found in these studies included epidermal skin burns and slight thermal injuries to the pectoralis major muscle post-MWA. In our case, the 1% lidocaine administered to our patient formed an isolation belt, and similar to Zhou et al.'s study [15], there were no epidermal burns. Our study's initial axillary lymph node ablation was incomplete due to significant discomfort and shoulder joint pain. However, the re-ablation of the lesion after two months was successful without recurrence and had excellent cosmesis. MRI follow-ups of the primary and axillary lymph node lesions showed tumor responses to ablations and significant reductions in the size of the ablated lesions.

The MWA system's frequency, energy delivery, and the relative phase of the alternating fields produced by antennas can all greatly impact the size of the resulting ablation zone [16,17]. Similar to a previous study [18], the primary lesion ablated was <3 cm, and an accurate placement of one antenna was required for a complete ablation. Our experience with MWA under ultrasound guidance is based on the use of the TATOpro's thinnest antenna (18G) in delivering a precise ablation for breast lesions.

## Conclusions

This case report demonstrates the successful use of TATOpro's MWA system for treating breast cancer in an 83-year-old patient in Africa. The absence of disease recurrence one year post-treatment, highlighted by the indispensable role of imaging, demonstrates MTA's potential as a viable, minimally invasive alternative to tumor resection. The significance of this case lies in its implications for patient-specific treatment strategies and its potential impact in resource-limited healthcare settings. It underscores the necessity for broader research, including larger studies and long-term follow-ups, to validate MTA's efficacy and safety in diverse patient populations.
